# Silencing of USP22 promotes FGF11 degradation to attenuates renal fibrosis in diabetic kidney disease

**DOI:** 10.1080/0886022X.2026.2668134

**Published:** 2026-06-10

**Authors:** Zhigang Wang, Chao Liu, Jing Lv, Jiping Sun, Xiaoyang Yu, Huixian Li

**Affiliations:** Department of Nephrology, Kidney Hospital, the First Affiliated Hospital of Xi’an Jiaotong University, Xi’an, China

**Keywords:** Diabetic kidney disease, FGF11, USP22, deubiquitination, fibrosis, mesangial cell

## Abstract

**Objectives:**

Diabetic kidney disease (DKD) is a major cause of end-stage renal disease that is characterized by renal fibrosis. Fibroblast growth factor 11 (FGF11) is reported to be involved in mesangial cell fibrosis; however, its mechanism is not fully understood. This study aimed to investigate the role of FGF11 and its ubiquitination regulatory mechanism mediated by ubiquitin-specific protease 22 (USP22) in DKD. SV40 MES 13 cells were treated with high glucose (HG).

**Methods:**

A DKD mouse model was established using streptozotocin injection and high-fat diet feeding (6 mice/group). FGF11 mRNA and protein levels in cells and kidneys of mice were detected using qPCR and immunoblotting. Cell proliferation was evaluated using cell counting kit-8 (CCK-8) and 5-ethynyl-2’-deoxyuridine (EdU) assay, and fibrosis was evaluated using immunoblotting. The regulation of USP22 on FGF11 ubiquitination was analyzed using immunoprecipitation and immunoblotting. Renal injury was assessed by measuring histological fibrosis and injury markers.

**Results:**

FGF11 was highly expressed in the kidneys of DKD mice and HG-treated cells. Knockdown of FGF11 inhibited HG-induced proliferation and fibrosis. Furthermore, we discovered that USP22 knockdown promoted FGF11 degradation by enhancing its K48-linked ubiquitination, thus reducing FGF11 stability. Overexpression of FGF11 reversed the inhibition of proliferation and fibrosis caused by USP22 silence. *In vivo*, USP22 knockdown alleviated renal injury and fibrosis in DKD mice *via* decreasing FGF11 expression.

**Conclusions:**

Silencing of USP22 inhibits mesangial cell proliferation and fibrosis by promoting the ubiquitination of FGF11, which is related to the alleviation of DKD, suggesting that targeting the USP22-FGF11 axis may provide a therapeutic strategy for DKD.

## Introduction

Diabetic Kidney Disease (DKD) is a debilitating complication of diabetes, affecting 25%-40% of diabetic patients [1,2]. The prevalence of DKD remains high due to the increased incidence of type 2 diabetes. It is characterized by extracellular matrix accumulation, glomerular hypertrophy, mesangial expansion, and eventually renal fibrosis, leading to chronic kidney disease and end-stage renal disease [3,4]. At present, treatments for DKD rely only on renin-angiotensin system inhibitors, dialysis, or kidney transplantation, but there is still no cure [5,6]. Thus, it is necessary to completely understand the underlying molecular mechanisms in order to identify novel therapeutic targets.

The fibroblast growth factor (FGF) family, consisting of 23 sequentially and structurally similar members, is involved in various important pathophysiological processes, such as embryonic development, wound healing, metabolism, and endocrinology [7]. FGF11 is a member of this family, has been implicated in numerous cellular biological processes, such as growth, differentiation, migration, and invasion, and thus acts as a prognostic marker or therapeutic target [8–10]. It is a type of intracellular FGFs, located in the nucleus or cytoplasm, and functions independently of FGF receptors within cells [10,11]. A recent study has suggested that FGF11 may play a role in the pathogenesis of diabetes [12]. Notably, FGF11 is involved in the pathogenesis of DKD, as it can regulate mesangial cell proliferation and fibrosis [13]. However, the regulatory mechanisms controlling FGF11 expression in DKD remain poorly understood.

Ubiquitination, due to its profound influence on the stability and activity of substrate proteins, has received more and more attention in the development of diseases [14]. It is a covalent post-translational modification of protein with ubiquitin that is modulated by E1 ubiquitin-activating enzyme, E2 ubiquitin-conjugating enzyme, and E3 ligase [15]. In addition, deubiquitinating enzymes (DUBs), also known as ubiquitin hydrolases, can remove ubiquitin from the substrates that play a critical role in degradation-independent ubiquitination [16,17]. It has been demonstrated that DUBs are involved in various diseases, such as cancers and neurodegenerative disorders. Thus, DUBs are promising therapeutic targets for the treatment of diseases; however, off-target effects pose a significant challenge in the treatment targeting DUBs [16]. Among them, the ubiquitin-specific protease (USP) family is the largest subfamily of DUBs that comprises 58 members with the most varied structure [18]. USP22 has been known to regulate protein stability through deubiquitination and is involved in the pathogenesis of several diseases, such as malignancy, metabolic disorder, and cardiovascular disease [19–21]. In DKD, USP22 has been reported to control renal tubulointerstitial fibrosis in a seminal study by Zhao et al. [22], which demonstrates that USP22 aggravates tubulointerstitial fibrosis by promoting epithelial-mesenchymal transition of renal tubular epithelial cells. However, the specific impact of USP22 on mesangium in the context of DKD and its potential relationship with FGF11 remain largely unexplored.

In this study, we aimed to investigate the role of FGF11 in the pathogenesis of DKD and to elucidate the upstream regulatory mechanisms involving USP22. We hypothesized that USP22 may regulate the deubiquitination of FGF11, thereby influencing the proliferation and fibrotic response of mesangial cells under hyperglycemic conditions, and thereby affecting DKD progression. This study may reveal a novel regulatory axis involving USP22 and FGF11 that contributes to the pathogenesis of DKD.

## Materials and methods

### Cell culture and stimulation

Mouse glomerular mesangial cell line (SV40 MES 13) and HEK-293T cells were purchased from ATCC (Manassas, VA, USA). The cells were maintained in Dulbecco’s modified Eagle’s medium (DMEM; Procell, Wuhan, China) plus 10% fetal bovine serum (FBS; Procell) and 1% Penicillin–Streptomycin (Procell) at 37 °C with 5% CO_2_.

The cells were divided into the control and high glucose (HG) groups. The cells in the control group were cultured in a medium containing low sugar (5.5 mM D-glucose) for 48 h, and the cells in the HG group were cultured in a medium containing high sugar (30 mM D-glucose) for 48 h.

### Cell transfection

SV40 MES 13 cells were inoculated in 6-well plates and grown until cell confluence was over 70%. FGF11 short hairpin RNA (shFGF11), shUSP22, shUSP9X, shTRIM13, shTRIM18, shTRIM29, shTRIM63, and negative control short hairpin RNA (shNC) plasmids, as well as FGF overexpression vectors and empty vectors, were constructed. These plasmids were transfected into SV40 MES 13 cells using Lipofectamine 2000 (Invitrogen, Carlsbad, CA, USA) for 48 h.

To assess FGF11 ubiquitination, HA-UB, HA-UB-K48R, HA-UB-K48, and HA-UB-K63 plasmids were purchased from Kelei Biology (Shanghai, China). USP22 C185A plasmid were synthesized by Biosen (Shanghai, China). HEK-293T cells were transfected with these plasmids as well as Flag-USP22 and His-FGF11 using Lipofectamine 2000.

### Quantitative real-time polymerase chain reaction (qPCR)

Total RNA was extracted using the TRIzol reagent (Invitrogen). After detecting RNA concentration using the NanoDrop2000 spectrophotometer (Thermo Fisher Scientific, Waltham, MA, USA), 1 μg RNA was used for reverse transcription at 55 °C for 5 min using the Hifair^®^ AdvanceFast 1st strand cDNA synthesis kit (Yeasen, Shanghai, China). Next, the cDNA was used as the template for qPCR using the Hieff^®^ qPCR SYBR Green Master Mix (Yeasen) on the CFX96 system (Bio-Rad). The expression of mRNAs was calculated using the 2^−ΔΔCt^ method. β-actin served as the internal control.

### Cell counting kit-8 (CCK-8)

Transfected SV40 MES 13 cells were inoculated into 96-well plates and treated with HG. Then, 10 μL CCK-8 solution (Dojingdo, Kumamoto, Japan) was incubated with the cells for another 2 h. Absorbance was read using the iMark microplate reader (Bio-Rad, Hercules, CA, USA) at 450 nm.

### EdU assay

Transfected SV40 MES 13 cells (5 × 10^4^ cells) were inoculated into 96-well plates and treated with HG. The Yefluor 594 EdU kit (Yeasen) was used to evaluate cell proliferation. The cells were labeled with 10 μM EdU working solution. After that, the cells were fixed with 50 μL 4% paraformaldehyde for 30 min, and 2 mg/mL glycine was incubated with the cells to neutralize residual paraformaldehyde. The cells were permeated with 0.1 mL 0.5% Triton X-100 for 20 min. Click-iT reaction mixture was prepared by Click-iT EdU reaction buffer, CuSO4, Yefluor 594 Azide, and EdU buffer additive. The cells were incubated with the Click-iT reaction mixture for 30 min in the dark. After staining DNA with DAPI for 20 min, the results were photographed under the LSM880 laser scanning confocal microscope (Carl Zeiss, Jena, Germany).

### Immunoblotting

To detect protein levels, SV40 MES 13 cells and the kidney tissues of mice were lysed using the radio-immunoprecipitation assay buffer (Beyotime, Shanghai, China). To detect FGF11 ubiquitination levels, SV40 MES 13 or HEK-293T cells were treated with 10 μM MG132 (MCE, Monmouth Junction, NJ, USA) 6 h before cell lysis. Following measuring protein concentration using a BCA protein assay kit (Sorlarbio, Beijing, China), 40 μg proteins were separated using 4%-12% sodium dodecyl sulfate-polyacrylamide gel electrophoresis (SDS-PAGE) and transferred onto polyvinylidene fluoride (PVDF) membranes (Millipore, Billerica, MA, USA). Following blockade, primary antibodies were incubated with the membranes at 4 °C overnight, and the secondary antibody was incubated with the membranes at 25 °C for 1 h. The bands of proteins were improved using the Super electrochemiluminescence (ECL) detection reagent (Yeasen). The results were quantified using the ImageJ software.

The primary antibodies included anti-FGF11 (PA5-103408, Invitrogen), anti-collagen 4 (Col-4; 14-9871-82, Invitrogen), anti-Fibronectin (sc-8422, Santa Cruz Biotechnology, Santa Cruz, CA, USA), anti-TGF-β1 (sc-130348, Santa Cruz Biotechnology), anti-β-actin (sc-56459, Santa Cruz Biotechnology), anti-USP22(sc-390585, Santa Cruz Biotechnology), anti-USP9X (ab19879, Abcam, Cambridge, MA, USA), anti-TRIM13 (ab194477, Abcam), anti-TRIM18 (sc-293353, Santa Cruz Biotechnology), anti-TRIM29 (ab244380, Abcam), anti-TRIM63 (sc-134397, Santa Cruz Biotechnology), anti-ubiquitin (UB; 13–1600, Invitrogen), anti-HA (ab18181, Abcam), anti-His (ab18184, Abcam), anti-Flag (ab125243, Abcam). The secondary antibodies included HRP-labeled goat anti-mouse (ab205719, Abcam) and HRP-labeled goat anti-rabbit (ab6721, Abcam).

### Immunoprecipitation (IP) and co-IP

SV40 MES 13 cells or HEK-293T cells were lysed using the IP lysis buffer, and the lysate was collected after centrifuging at 14000 ×g for 10 min. Cell lysate was incubated with 5 μg antibodies at 4 °C overnight, and incubated with Protein A/G magnetic beads at 4 °C for 3 h. The sediments were collected after centrifuging at 12000 ×g for 1 min and washed with washing buffer. The samples were evaluated using immunoblotting. The antibodies used for IP included anti-FGF11 (PA5-103408, Invitrogen), anti-USP22 (sc-390585, Santa Cruz Biotechnology), anti-IgG (ab172730, Abcam), and anti-His (ab213204, Abcam).

### Protein stability determination

SV40 MES 13 cells after shNC and shUSP22 transfection were treated with 50 μg/mL cycloheximide (CHX; MCE) for the indicated times (0, 8, 16, and 24 h). Protein was extracted, and FGF12 levels were detected using immunoblotting.

### Animal experiments

The animal study was conducted following the Guide for the Care and Use of Laboratory Animals and ARRIVE guidelines. A total of 36 C57BL/6J mice (6-week-old, half male and half female) were purchased from Cavens (Changzhou, China) and housed in cages at 20–22 °C, 12-h light/dark cycles, 55% humidity with free access to food and water. The mice were divided into six groups, including control (CON), DKD, DKD + lentivirus (Lv)-shNC, DKD + Lv-shUSP22, DKD + Lv-shUSP22 + Lv-NC, and DKD + Lv-shUSP22 + Lv-FGF11, with six mice per group. Mice were grouped according to the randomization formula. Streptozotocin (STZ) was purchased from Sigma-Aldrich (St. Louis, MO, USA) and dissolved in 0.1 M pH 4.5 sodium citrate buffer. To induce the mouse model, mice were fed a high-fat diet (60% calories from fat) for 2 weeks, and injected with 50 mg/kg STZ for 5 days, once a day. The mice with fasting blood glucose higher than 300 mg/dl were considered diabetic mice. These mice were continued to be fed a high-fat diet. The mice in the control group were fed a normal diet and received an equal amount of sodium citrate buffer injection. Two weeks later, the model was confirmed again by detecting fasting blood glucose. The mice in the DKD + Lv-shNC, DKD + Lv-shUSP22, DKD + Lv-shUSP22 + Lv-NC, and DKD + Lv-shUSP22 + Lv-FGF11groups were injected with lentivirus (1 × 10^8^ TU/mL) through the tail vein, and the mice in the other two groups were injected with PBS. After 4 weeks, the urine of mice collected within 24 h was analyzed, and proteinuria was detected. Then, the mice were fasted overnight, and blood glucose was measured. To measure FGF11 ubiquitination levels, mice were intraperitoneal injected with 2.5 mg/kg MG132 6 h before euthanasia. Finally, mice were sacrificed by inhaling isoflurane. The abdominal aorta blood sample and kidneys were collected. Serum creatinine and blood urea nitrogen were measured by an automatic biochemical analyzer. The kidney tissues were used for hematoxylin and eosin (HE) and Masson’s trichrome staining. The investigators involved in the assessment of physiological indicators and the evaluation of histological sections were blinded to the group allocation. Tubular injury was scored as previously described [23]. The fibrosis area was quantified using the Image-Pro Plus software. This study was approved by the Ethics Committee of Health Science Center of Xi’an Jiaotong University (No. XJTUAE2024-2372).

### Statistical analysis

Data were analyzed using the GraphPad Prism 7 software. The normality of the data distribution was assessed using the Kolmogorov–Smirnov test, and all data conformed to normal distribution. Differences between the two groups were assessed for significance using the unpaired t-test. For comparisons among more than two groups with normal distribution and equal variance (assessed by the Brown-Forsythe test), one-way analysis of variance (ANOVA) was performed, followed by Tukey’s *post hoc* test for multiple comparisons. The differences in cell viability and the stability of FGF11 protein were analyzed using two-way ANOVA followed by a Bonferroni *post hoc* test. Statistically significant was considered when *p* < 0.05.

## Results

### FGF11 expression is upregulated in DKD mice and HG-induced cells

**Figure 1.**
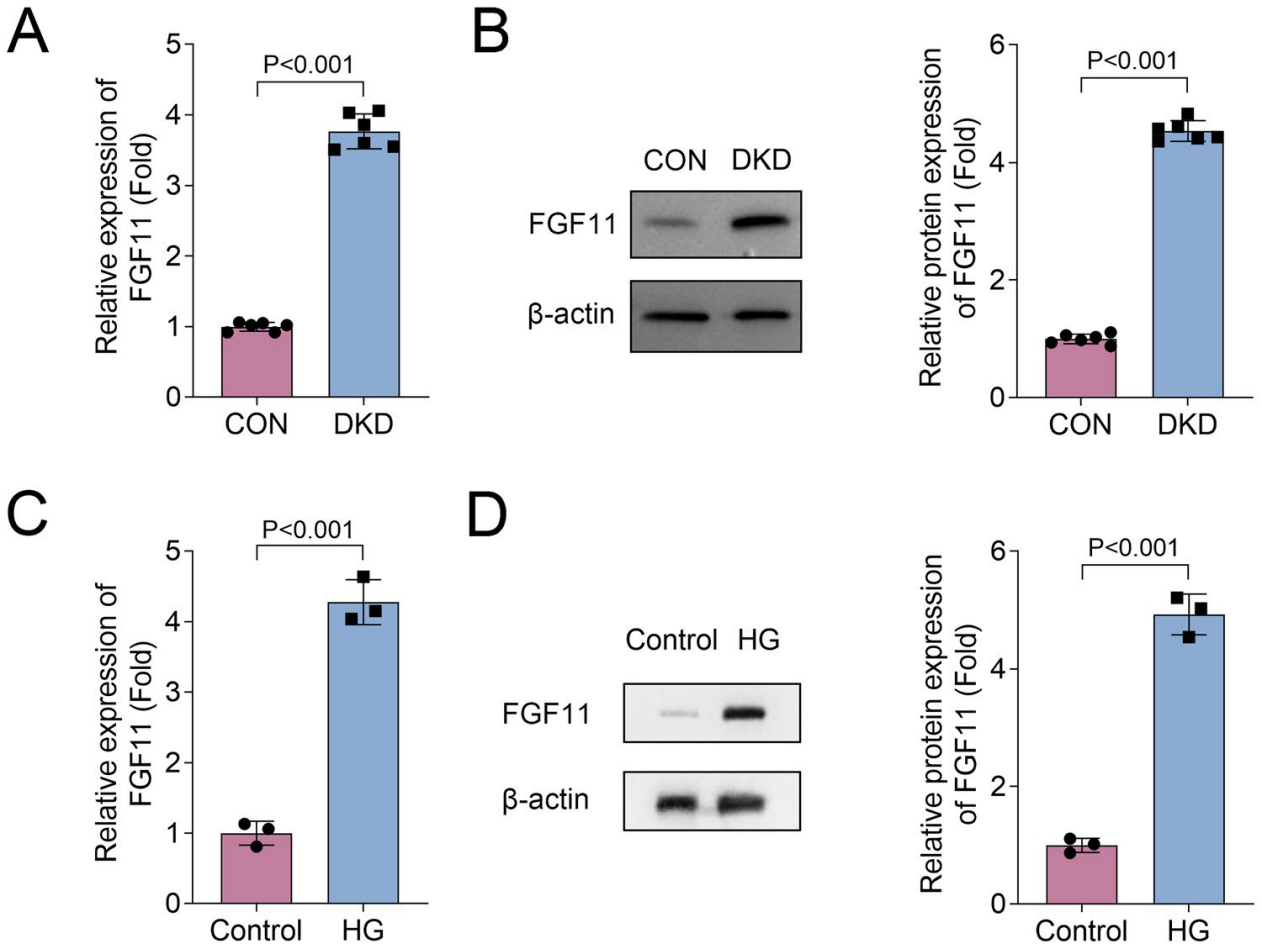
FGF11 expression is upregulated in DKD mice and HG-induced cells. The (A) mRNA and (B) protein levels of FGF11 in the kidneys of mice in the control and DKD groups were measured using qPCR and immunoblotting, respectively. Six biological replicates for each group. The (C) mRNA and (D) protein levels of FGF11 in SV40 MES 13 cells treated with HG or not were measured using qPCR and immunoblotting, respectively. Three biological replicates for each group.

To investigate the potential role of FGF11, we first generated a DKD mouse model and measured its expression in the kidneys. We found that the expression of FGF11 was elevated in the kidney tissues of DKD mice, compared with the control group mice (Figure 1A,B). Next, SV40 MES 13 cells were treated with HG to simulate cell damage caused by hyperglycemia. As expected, FGF expression was upregulated in HG-induced SV40 MES 13 cells, compared with untreated cells (Figure 1C,D). The results suggest that FGF11 plays a role in the progression of DKD, particularly affecting mesangial cell processes.

### Knockdown of FGF11 suppresses the proliferation and fibrosis of HG-induced cells

**Figure 2.**
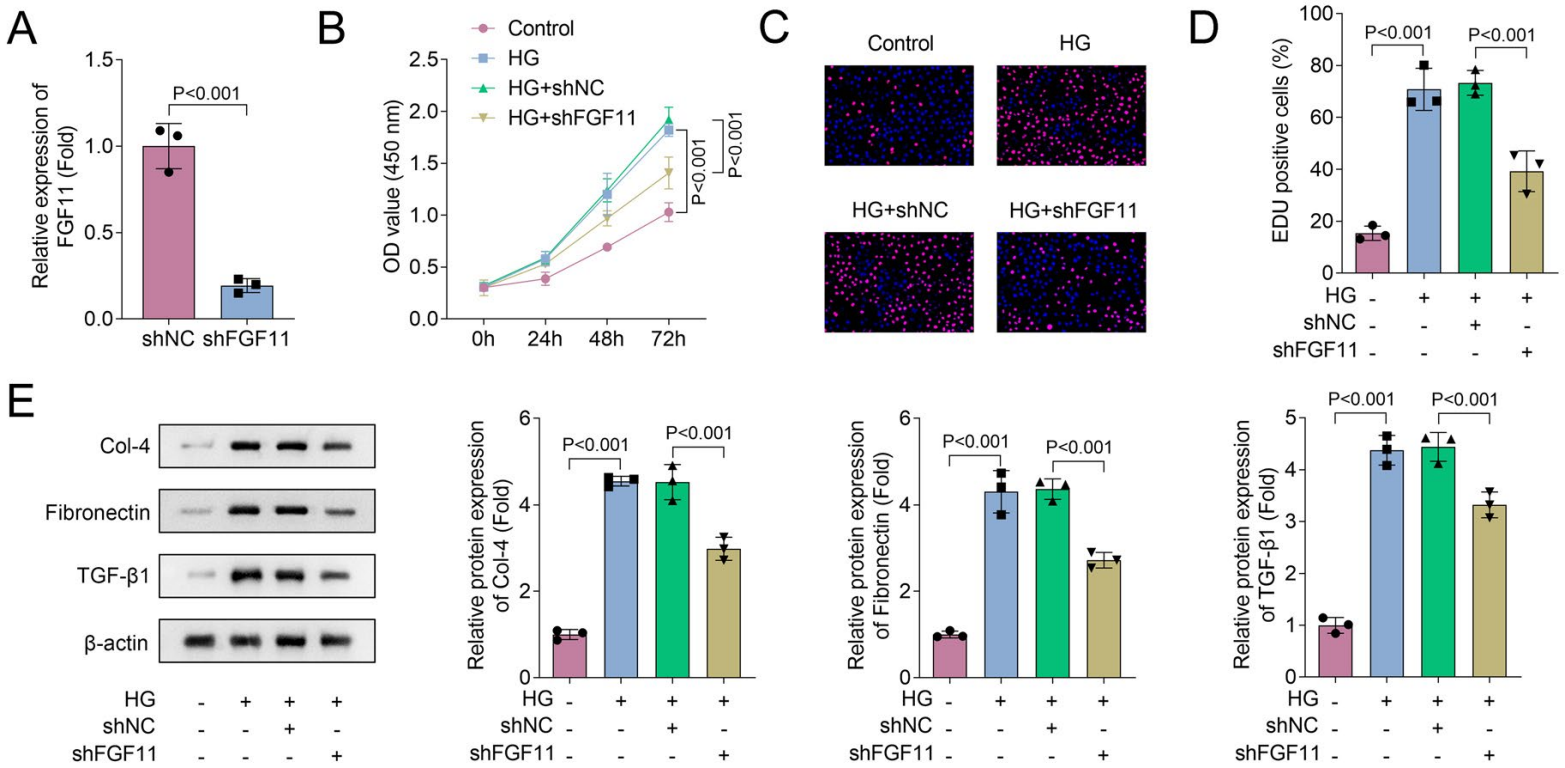
Knockdown of FGF11 suppresses the proliferation and fibrosis of HG-induced cells. (A) SV40 MES 13 cells were transfected with shNC or shFGF11, and the expression of FGF11 was measured using qPCR. SV40 MES 13 cells were transfected with shNC or shFGF11, and treated with HG, and (B) cell viability was measured using CCK-8; (C, D) cell proliferation was analyzed using EdU assay, and EdU positive cells were counted; (E) the levels of Col-4, fibronectin, and TGF-β1 were detected using immunoblotting. Three biological replicates for each group.

To explore how FGF11 affected SV40 MES 13 cell biological behaviors, we transfected shFGF11 and shNC into the cells. Following transfection of shFGF11, FGF11 expression was reduced, compared with shNC transfection (Figure 2A). Subsequently, cell viability was assessed using CCK-8 assay. The results showed that HG enhanced cell viability, while FGF11 knockdown suppressed the viability of HG-induced cells (Figure 2B). Besides, EdU assay was performed to conduct cell proliferation. We found that the promotion of the proliferation caused by HG was reversed by FGF11 silence (Figure 2C,D). In addition, fibrotic response was evaluated by detecting the levels of several fibrosis-related factors. Immunoblotting results showed that the levels of Col-4, fibronectin, and TGF-β1 were increased in HG-treated cells, which was counteracted by knockdown of FGF11 (Figure 2E). The results demonstrated that HG promotes SV40 MES 13 cell proliferation and fibrosis dependent on the upregulation of FGF11 expression.

### USP22 is necessary for the deubiquitination of FGF11

**Figure 3.**
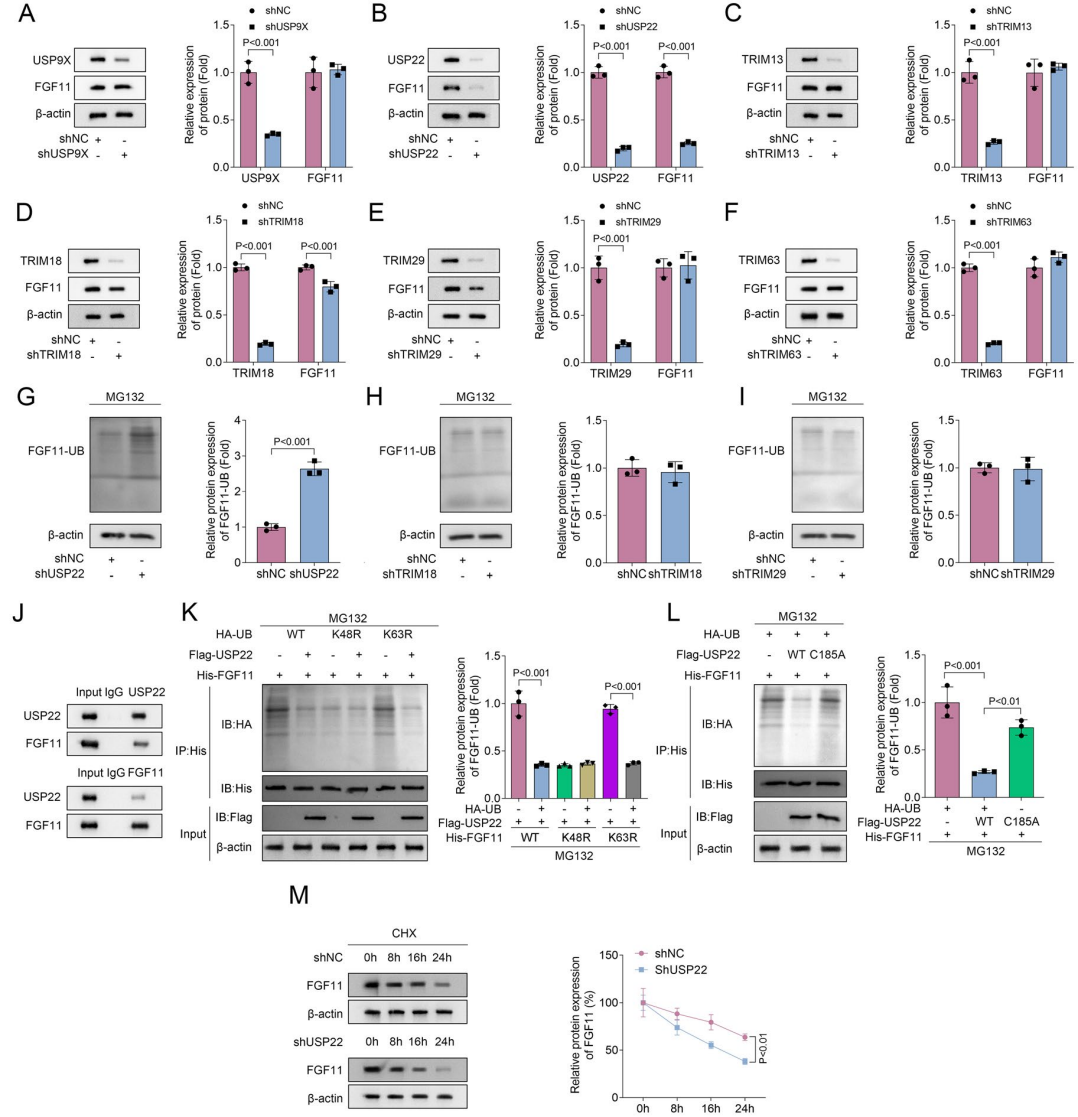
USP22 is necessary for the deubiquitination of FGF11. The effect of several DUBs, including (A) USP9X, (B) USP22, (C) TRIM13, (D) TRIM18, (E) TRIM29, and (F) TRIM63, on the protein levels of FGF11 was evaluated using immunoblotting. The impact of (G) USP22, (H) TRIM18, and (I) TRIM29 on the ubiquitination levels of FGF11. (J) The interaction between USP22 and FGF11 proteins was analyzed using co-IP. (K) HEK-293T cells were transfected with Flag-USP22, His-FGF11, and WT/K48R/K63R HA-UB. After IP with His, immunoblotting was performed with anti-HA after MG132 treatment. (L) HEK-293T cells were transfected with HA-UB, His-FGF, and WT USP22 or USP22 C185A. After IP with His, immunoblotting was performed with anti-HA after MG132 treatment. (M) SV40 MES 13 cells were transfected with shNC or shUSP22 and treated with CHX for 0, 8, 16, and 24 h. Protein levels of FGF11 were detected using immunoblotting. Three biological replicates for each group.

To identify which DUB could affect FGF11 degradation, we chose several enzymes that have been reported to be involved in DKD progression. The effect of these enzymes on FGF11 protein levels was first assessed. As shown in Figure 3A–F, the levels of FGF11 were downregulated after knocking down USP22 and TRIM18. Additionally, silencing of USP9X, TRIM13, TRIM29, and TRIM63 did not change FGF11 protein levels. Subsequently, we evaluated the regulation of USP22, TRIM18, and TRIM29 on FGF11 ubiquitination. It was observed that only USP22 knockdown enhanced the ubiquitination levels of FGF11, while TRIM18 and TRIM29 did not affect FGF11 ubiquitination (Figure 3G–I). Hence, USP22 was chosen for further study. The results of co-IP showed that the USP22 antibody could IP FGF11, and the FGF11 antibody could IP USP22 (Figure 3J), suggesting that USP22 interacts with FGF11. SV40 MES 13 cells were transfected with shNC or shUSP22 and treated with MG132. After IP with IgG antibody, no ubiquitination bands were observed; however, FGF11 ubiquitination levels were higher in the shUSP22 group than that in the shNC group when using the FGF11 antibody for IP (Figure S1). The results suggest that the ubiquitination signal is associated with FGF11 rather than nonspecific background. Then, we investigated the effects of USP22 on two common polyubiquitin linkages, K48 and K63 of FGF11. HEK-293T cells were transfected with HA-UB (WT, K48R, or K63R), Flag-USP22, and His-FGF11. After IP with His antibody, immunoblotting was performed to detect ubiquitination levels of FGF11 after MG132 treatment. We found that USP22 decreased the ubiquitination levels of FGF11 in the WT and K63R group, but did not affect them when the K48 linkage was mutated (Figure 3K), indicating that USP22 promotes K48-linked ubiquitination of FGF11. To further confirm that ubiquitination occurs on the K48-linked ubiquitin chain, HEK-293T cells were transfected with HA-UB (WT or K48), Flag-USP22, and His-FGF11. The results showed that USP22 reduced FGF11 K48-linked ubiquitination (Figure S2). To confirm that ubiquitination depends on the deubiquitinating enzyme activity of USP22, an enzyme-dead mutant (USP22 C185A) was constructed [24]. The results of immunoblotting showed that WT USP22 suppressed the ubiquitination levels of FGF11, whereas USP22 C185A increased the ubiquitination levels of FGF11, compared with the WT group (Figure 3L). Moreover, because K48-linked ubiquitination is associated with protein degradation, we analyzed the protein stability of FGF11 mediated by USP22 in the presence of CHX. The results showed that knockdown of USP22 reduced the protein stability of FGF11 (Figure 3M), suggesting that USP22 stabilizes FGF11 through the ubiquitination mechanism. Taken together, USP22 inhibits K48-linked ubiquitination of FGF11 and stabilizes FGF11 protein.

### Knockdown of USP22 suppresses HG-induced proliferation and fibrosis by decreasing FGF11 expression

**Figure 4.**
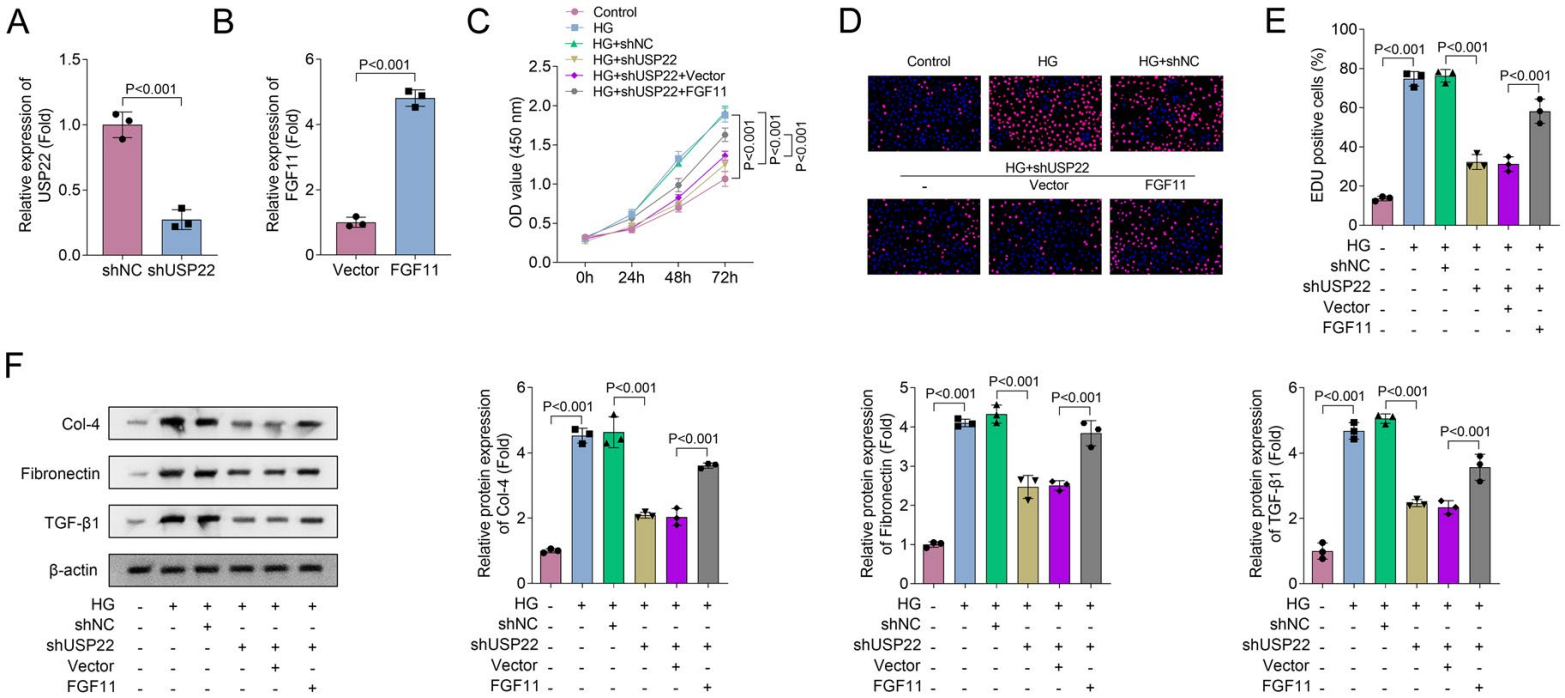
Knockdown of USP22 suppresses HG-induced proliferation and fibrosis by decreasing FGF11 expression. (A) SV40 MES 13 cells were transfected with shNC or shUSP22, and the expression of USP22 was measured using qPCR. (B) SV40 MES 13 cells were transfected with FGF11 overexpression plasmids or empty vectors, and the expression of FGF11 was measured using qPCR. After knocking down USP22 and overexpressing FGF11, SV40 MES 13 cells were treated with HG, and (C) cell viability was measured using CCK-8; (D, E) cell proliferation was analyzed using EdU assay, and EdU positive cells were counted; (F) the levels of Col-4, fibronectin, and TGF-β1 were detected using immunoblotting. Three biological replicates for each group.

Based on the regulation of USP22 on FGF11, the impacts of the USP22/FGF11 axis on the proliferation and fibrotic response of SV40 MES 13 cells were assessed. Following shUSP22 transfection, the expression of USP22 was reduced, compared with shNC transfection (Figure 4A). Additionally, FGF11 expression was upregulated after its overexpression plasmid transfection (Figure 4B). Silencing of USP22 reduced cell viability and EdU positive cells after HG treatment, whereas FGF11 overexpression reversed this reduction (Figure 4C–E), suggesting that FGF11 reversed the inhibition of cell proliferation induced by USP22 knockdown. Additionally, the downregulation of Col-4, fibronectin, and TGF-β1 levels mediated by USP22 knockdown was abrogated by FGF11 overexpression (Figure 4F). In short, FGF11 can abrogate the inhibition of the proliferation and fibrosis of HG-treated SV40 MES 13 cells mediated by USP22 knockdown.

### Silencing of USP22 protects mice from renal injury and fibrosis by regulating FGF11 levels

**Figure 5.**
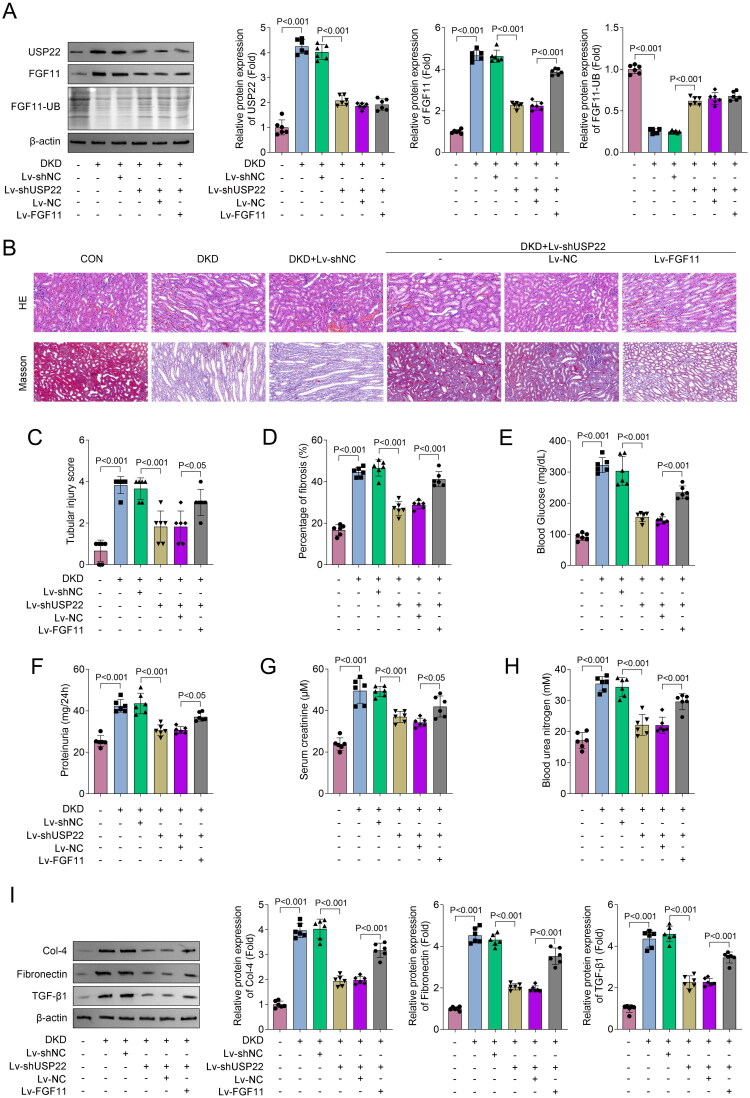
Silencing of USP22 protects mice from renal injury and fibrosis by regulating FGF11 levels. (A) USP22 and FGF11 protein levels and FGF11 ubiquitination levels were measured using immunoblotting. (B) Represent images of HE and Masson’s trichrome staining of the kidney of mice. (C) Tubular injury scores. (D) Quantification results of renal fibrosis. (E) The blood glucose, (F) 24 h proteinuria, (G) serum creatinine, and (H) blood urea nitrogen were detected. (I) Protein levels of Col-4, fibronectin, and TGF-β1 were measured in the kidney of mice. Six biological replicates for each group.

To explore the role of USP22 and FGF11 *in vivo*, we generated the diabetic mouse model to induce DKD and injected it with Lv-shNC, Lv-shUSP22, Lv-NC, and Lv-FGF11. The kidneys were collected from all mice. Immunoblotting results showed that USP22 and FGF11 protein levels were upregulated, and FGF11 ubiquitination levels were downregulated in the DKD groups, which was reversed after Lv-shUSP22 injection. Additionally, overexpression of FGF11 increased its own protein levels, but did not influence its ubiquitination levels and USP22 protein levels (Figure 5A). Additionally, the kidney tissue specimens were collected for HE and Masson’s trichrome staining. The HE staining results showed that the renal pathology in the model was worse than that in control mice, and the fibrosis level was higher than that in control mice, which were abrogated by USP22 knockdown; however, FGF11 overexpression reversed the effect of USP22 silence (Figure 5B–D). Several renal injury-related indicators were detected in mice in each group. As expected, the blood glucose, 24-h proteinuria, serum creatinine, and blood urea nitrogen were increased in DKD mice. USP22 silence reduced their levels in DKD mice, which was counteracted by FGF11 overexpression (Figure 5E–H). Moreover, the levels of Col-4, fibronectin, and TGF-β1 in the renal tissues were increased in DKD mice. Silencing of USP22 downregulated their levels, whereas FGF11 overexpression abrogated this downregulation (Figure 5I). Taken together, knockdown of USP22 attenuates DKD in diabetic mice by decreasing FGF11 levels.

## Discussion

Our study provides novel insights into the molecular mechanisms underlying the pathogenesis of DKD by identifying a critical role for the DUB USP22 in the regulation of FGF11 protein stability. This study expands on previous research by linking these two factors in the context of DKD, offering a new perspective on this disease.

The members of the FGF family have been reported to be dysregulated in DKD and are involved in DKD progression by regulating differentiation mechanisms. For example, FGF23 expression is increased in patients with DKD and is associated with endothelial dysfunction [25]. Liang et al. [26] have found that FGF1 expression is decreased in DKD, and the high FGF1 expression inhibits the inflammatory response of mesangial cells and podocytes. Li et al. [27] have revealed that FGF21 treatment downregulates fibrosis-related factors in mesangial cells by inhibiting ECM overexpression. FGF11 has been considered to affect several diseases, such as cancer, foot-and-mouth disease, and diabetes [12,28,29]. In diabetes, FGF11, as a target of miR-342-3p, affects endothelial cell proliferation and migration. However, there are few studies on the role of FGF11 in DKD, and only one study showed that FGF11 increases the proliferation rate and fibrotic response of mesangial cells [13]. Here, we found that FGF11 was overexpressed in the kidneys of DKD mice, suggesting that FGF11 may be involved in DKD progression. Abnormal proliferation of mesangial cells leads to excessive extracellular matrix production, which leads to decreased glomerular filtration rate, aggravating glomerular sclerosis and renal fibrosis [30]. Thus, exploring the molecular mechanism of mesangial cell regulation is important. In this study, we found that knockdown of FGF11 inhibited the proliferation and fibrosis of HG-induced mesangial cells. These findings are consistent with the previous study [13], suggesting FGF11 is a potential therapeutic target for DKD.

Furthermore, based on the important role of ubiquitination-modified proteins in DKD [31,32], we explored the mechanism of deubiquitination regulation of FGF11 from the perspective of ubiquitination. Our results demonstrate that knockdown of USP22 directly regulates FGF11 protein stability through the promotion of its K48-linked ubiquitination, leading to increased proteasomal degradation, consistent with previous studies showing that USP22 promotes protein stabilization [33,34]. These findings identify a novel post-translational regulatory mechanism for FGF11. It is known that USP22 plays a critical role in human diseases. This is due to the fact that USP22 regulates multiple cellular processes, including autophagy, proliferation, apoptosis, inflammation, and fibrosis [35–37]. DUBs commonly regulate protein function in situations of environmental change and stress [16]. Under HG, USP22 expression can increase to participate in diabetes [38]. Moreover, USP22 aggravates renal tubulointerstitial fibrosis by stabilizing Snail 1 [22]. Nevertheless, whether USP22 affects the biological behaviors of mesangial cells remains not understood. Remarkably, our study showed that knocking down USP22 inhibited mesangial cell proliferation and fibrosis, which can be reversed by FGF11 overexpression. Moreover, the animal experiments showed that silencing of USP22 alleviates DKD by suppressing renal injury and fibrosis. These findings suggest that USP22 is promising for therapeutic interventions in DKD.

It should be noted that our animal study employed a tail-vein injection of lentivirus to knock down USP22 and overexpression. We cannot fully rule out the potential contribution of extra-renal effects (i.e., off-target effects) to the observed renoprotective phenotype. Additionally, we have not demonstrated *in vivo* that the USP22/FGF11 axis directly targets mesangial cells. In our future work, we will adopt the method of injecting lentivirus into the kidneys and even a mesangial cell-specific USP22 knockout and FGF11 overexpression mouse model to assess the renal-specific role of USP22 and FGF11 in DKD.

In conclusion, our study reveals a novel regulatory mechanism of USP22 on FGF11 that contributes to the pathogenesis of DKD. We found that silencing of USP22 facilitates the degradation of FGF11 *via* the K48-linked ubiquitination, which suppresses the proliferation and fibrosis of mesangial cells, thereby alleviating DKD. Therefore, our findings reveal a novel pathogenic mechanism and suggest that the USP22-FGF11 axis could be a potential therapeutic target for DKD, warranting further investigation in more complex models and ultimately in clinical settings.

## Supplementary Material

S2.jpg

S1.jpg

## Data Availability

The datasets used and/or analyzed during the current study are available from the corresponding author on reasonable request.
