# Supplementary figures and images for: Silencing of USP22 promotes FGF11 degradation to attenuates renal fibrosis in diabetic kidney disease
Source: Ren Fail. 2026 Jun 10;48(1):2668134. doi: 10.1080/0886022X.2026.2668134 (PMC13255211; doi:10.1080/0886022X.2026.2668134)

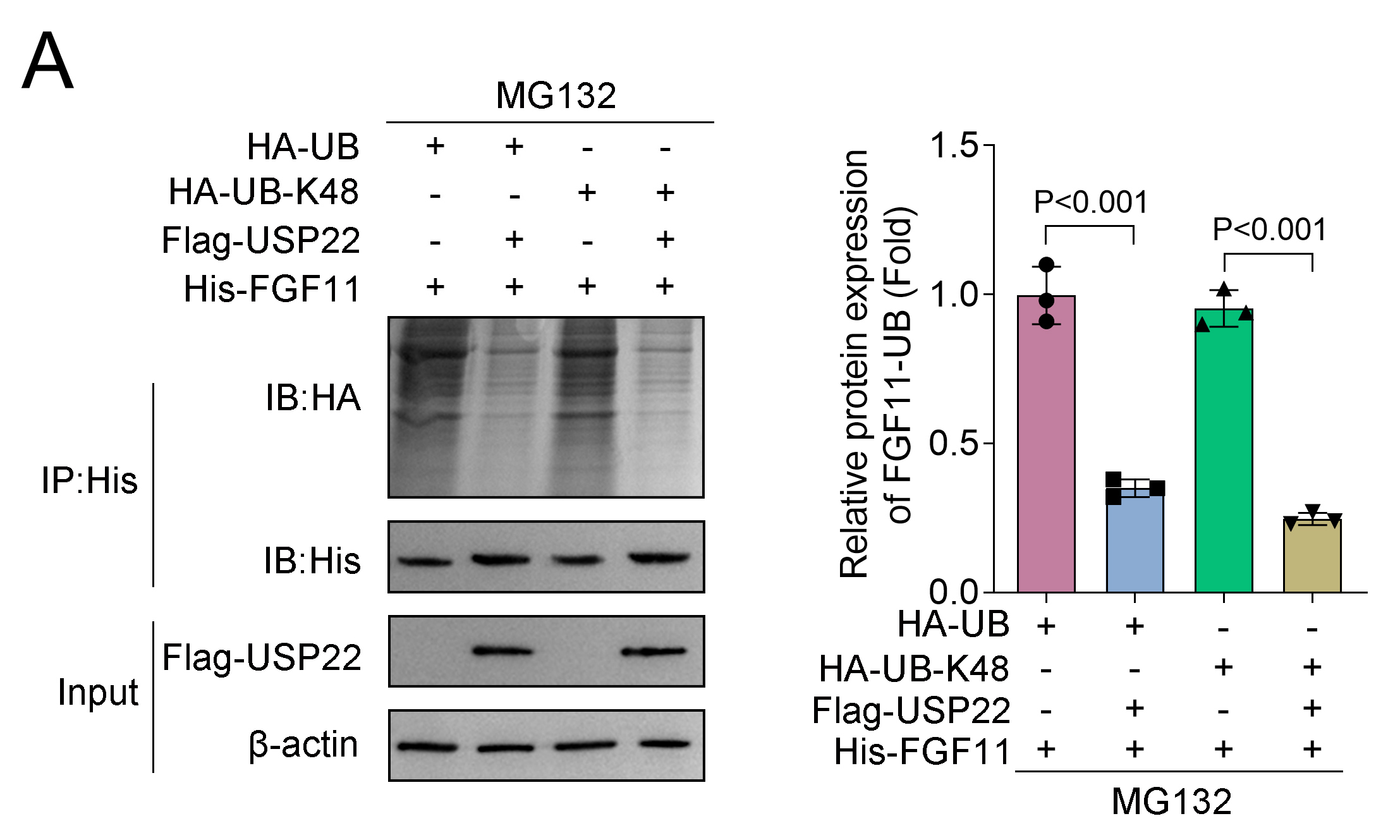

Supplement: S2.jpg [file IRNF_A_2668134_SM6137.jpg]

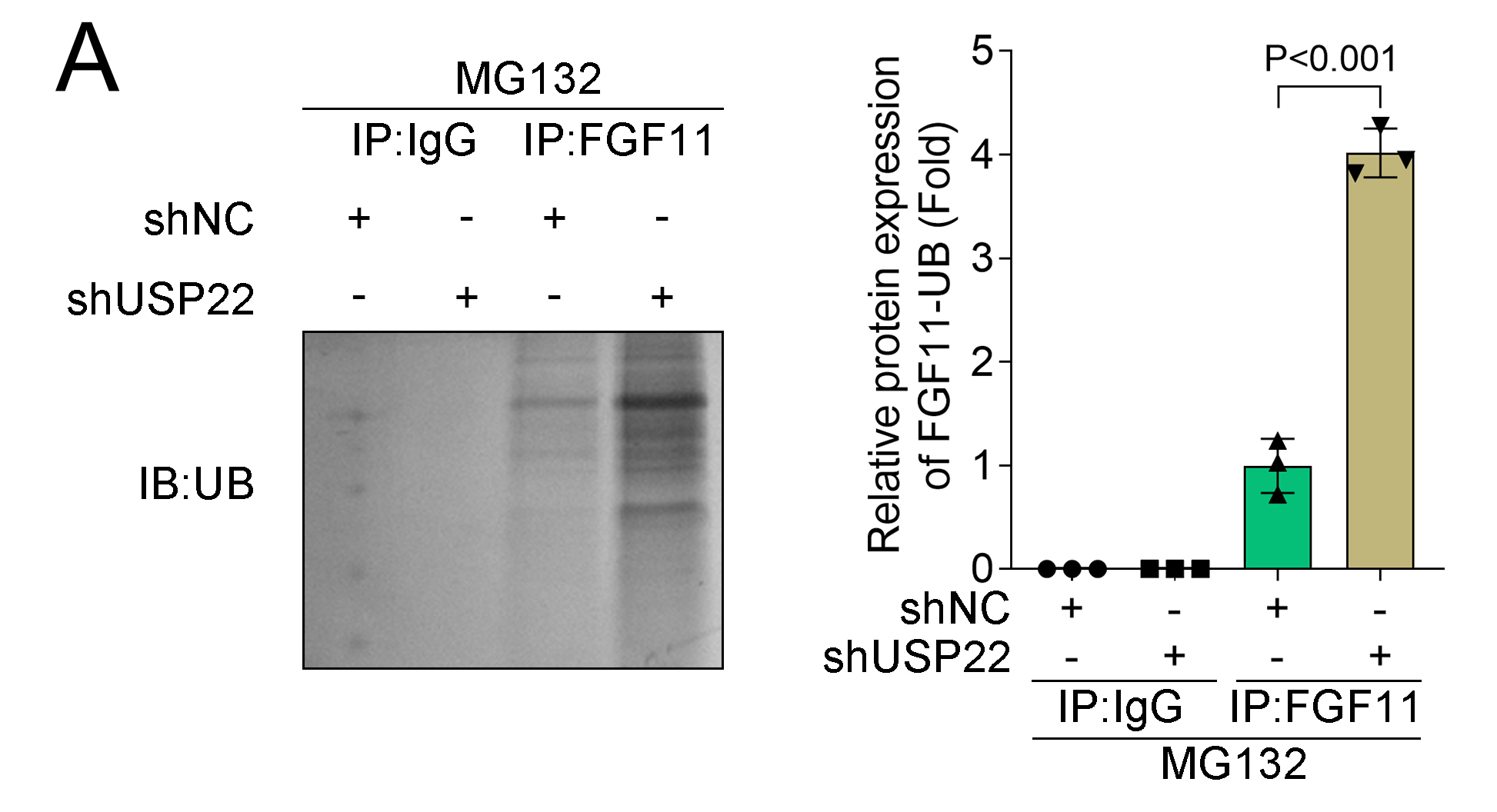

Supplement: S1.jpg [file IRNF_A_2668134_SM6136.jpg]
